# The Unique Karyotype of *Henochilus wheatlandii*, a Critically Endangered Fish Living in a Fast-Developing Region in Minas Gerais State, Brazil

**DOI:** 10.1371/journal.pone.0042278

**Published:** 2012-07-27

**Authors:** Priscilla C. Silva, Udson Santos, Natália M. Travenzoli, Jose C. Zanuncio, Marcelo de B. Cioffi, Jorge A. Dergam

**Affiliations:** 1 Laboratório de Sistemática Molecular - Beagle, Departamento de Biologia Animal, Universidade Federal de Viçosa, Viçosa, Brazil; 2 Laboratório de biodiversidade molecular e citogenética, Departamento de Genética e Evolução, Universidade Federal de São Carlos, São Carlo, Brazil; Virginia Tech, United States of America

## Abstract

*Henochilus wheatlandii*, the only species of this genus, is critically endangered and was considered extinct for over a century. The rediscovery of this fish in 1996 made it possible to study its phylogenetic relationships with other species in the subfamily Bryconinae. The aim of this study was to characterise the karyotype of *H. wheatlandii*. Standard staining, C-positive heterochromatin and nucleolar organiser region (NOR) banding, chromomycin A_3_ staining, and fluorescent *in situ* hybridisation (FISH) using 5S rDNA and 18S rDNA probes were conducted on nineteen specimens collected in the Santo Antonio River, a sub-basin of the Doce River in Ferros municipality, Minas Gerais State, Brazil. *Henochilus wheatlandii* shared the same diploid number and chromosome morphology as other species of Bryconinae. However, its heterochromatin distribution patterns, NOR localisation, and FISH patterns revealed a cytogenetic profile unique among Neotropical Bryconinae, emphasizing the evolutionary uniqueness of this threatened species.

## Introduction

*Henochilus wheatlandii* is the only species of this genus. It was first collected by the Thayer expedition in 1865 and 1866. The first record was from the Mucuri River Basin [1], an isolated drainage area in eastern Brazil. The second record did not provide details of its location [2]. *Henochilus wheatlandii* was described in 1890 [3], but collection efforts in this type locality during the late 20^th^ century were unsuccessful. Therefore, this species was officially considered to be at risk or extinct [4], [5]. In 1996, *H. wheatlandii* was collected in Preto do Itambé River, a tributary of the Santo Antonio River of the Doce River Basin in Minas Gerais State, Brazil. Based on this finding, the absence of new captures in the Mucuri River Basin was considered a record error of the type locality of the holotype [6], and the current distribution of *H. wheatlandii* now includes the Santo Antonio River Basin. Alternatively, this species could have become extinct in the type locality [6].

The systematics of *H. wheatlandii* have always been a matter of debate, and different authors have placed the species in different subfamilies [2], [7], [8]. Today, this genus is considered a member of the subfamily Bryconinae [9]. The close phylogenetic relationship of *H. wheatlandii* with members of this subfamily was corroborated with molecular [1], [10] and morphological data analyses [1]. The patterns obtained for the 16S ribosomal mitochondrial gene suggested a paraphyletic condition of the genera *Brycon* within Bryconinae [10], where *Brycon insignis* and *Brycon ferox* are more closely related to *H. wheatlandii* than other *Brycon*. Morphologically, the most evident difference between *Henochilus* and *Brycon* is the arrangement of premaxillary teeth in a double row in *Henochilus* and three rows in *Brycon* [1], a character variation that has been considered insufficient to justify the existence of the genus *Henochilus* [10].

Bryconins are indicators of high quality environmental habitat because they require rapid flowing waters with sandy substrate, highly oxygenated water, riparian vegetation and a moderate amount of nutrients [11]. These habitat conditions also restrict the distribution of *H. wheatlandii* to the Santo Antonio River Basin [6], although predatory exotics may also be a limiting factor for the presence of this species in the Doce River. Unfortunately, this species was rediscovered in a drainage area currently threatened by human activities such as mining, logging and hydroelectric projects [12].

Cytogenetic descriptions of Bryconinae are restricted to eight species that occur in the Amazon, São Francisco and Paraná watersheds [13] and one species in the Paraíba do Sul drainage. Historically, cytogenetic data on Ostariophysan fish have allowed the identification of cryptic species [14], the characterization of populations [15], [16], and the formulation of phylogenetic and phylogeographic hypotheses [17]–[19]. This study reports the first cytogenetic data for *H. wheatlandii* and compares the results with other species of Bryconinae from a biogeographic and phylogenetic perspective.

## Materials and Methods

Nineteen individuals, six females, eleven males and two juveniles, were collected in the Santo Antonio River, a sub-basin within the Doce River Basin (S19°13′858″ W43°04′905″) in Ferros municipality, Minas Gerais State, Brazil. The specimens were transported to the laboratory and anaesthetised with clove oil [20]. Following dissection, mitotic chromosomes were obtained from cell suspensions of the anterior kidney, using the conventional air-drying method [21]. Chromosomes were analysed with silver nitrate staining [22] to visualise the nucleolar organiser regions (Ag-NOR) in addition to the standard Giemsa method. C-banding was used to detect C-positive heterochromatin [23]. Chromosome guanine-cytosine (GC) rich regions were identified using chromomycin A_3_ (CMA_3_) fluorescence staining [24]. FISH was performed according to Pinkel and colleagues [25] using 18S rDNA [26] and 5S rDNA [27] probes. The probes were labelled by nick translation with biotin-14-dATP. Signal detection and probe amplification were performed using conjugated avidin-fluorescein isothiocyanate (FITC) and anti-avidin-biotin, and the metaphases were analysed with an epifluorescence microscope. The chromosomal images were captured using CoolSNAP-Pro software. The chromosomes were measured using Image Pro Plus® and classified following Levan and colleagues [28]. Voucher specimens were deposited in the João Moojen de Oliveira Museum of Zoology in Viçosa, Minas Gerais State, Brazil (MZUFV3195, MZUFV3744, MZUFV3791, MZUFV3821, MZUFV3827, and MZUFV4011). Collecting permit SISBIO14975-1 was issued to Prof. Jorge A. Dergam.

## Results

**Figure 1:**
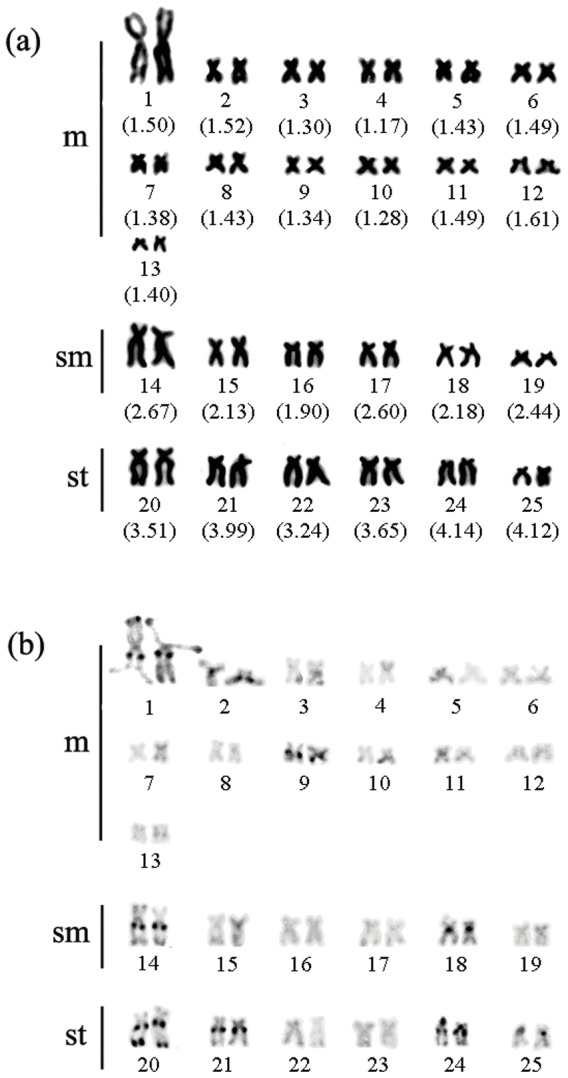
The karyotype of *Henochilus wheatlandii* from the Santo Antonio River. Conventional staining (Giemsa) (a) and heterochromatic marks obtained by C-banding protocol (b). Mean values of arm ratios are in parentheses (sample size = 40).

All specimens had 2n = 50 chromosomes and a karyotypic formula of 26m+12sm+12st (see Figure 1a and S1), with no differences observed between males and females. At least 40 Giemsa-stained, five C-banded, two Ag-NOR, two CMA_3_ and two FISH metaphases were analysed for each specimen. The mean values of the arm ratios were 1.28–1.61 (s.e. 0.002–0.2) for metacentrics; 1.90–2.67 (s.e. 0.05–0.2) for submetacentrics, and 3.24–4.14 (s.e. 0.04–0.2) for subtelocentrics. One pericentromeric and two telomeric heterochromatic blocks were visible in the largest pair of metacentric chromosomes of *H. wheatlandii*. Pericentromeric heterochromatin was evident in metacentric chromosome pairs 2 and 9 and submetacentric chromosome pairs 14 and 18, whereas the largest subtelocentric chromosomes (pair 20) presented conspicuous telomeric and pericentromeric heterochromatic blocks. Pericentromeric blocks predominated in most subtelocentrics, except for pairs 22 and 23 (see Figure 1b). At least five C-banded metaphases were analysed for each specimen.

**Figure 2:**
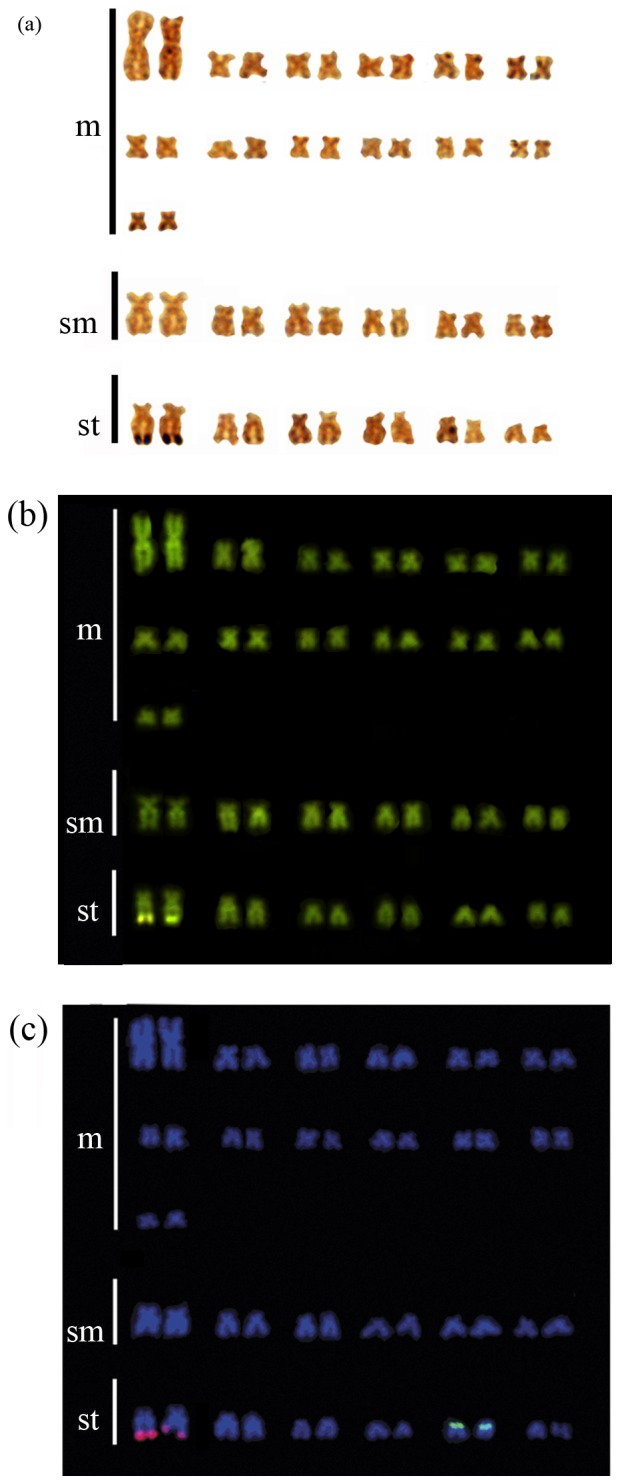
Cytogenetic marks in the same chromosome pair of *Henochilus wheatlandii* karyotype. Ag-NOR banding (a), chromomyicin A_3_ (b) and flourescent *in situ* hybridisation using 18S (pink) and 5S (green) probes (c).

Sites marked with Ag-NOR, CMA_3_, and 18S rDNA sites were telomeric and identified in the same first pair of subtelocentric chromosomes (see Figure 2). The 5S rDNA regions were distributed in the short arm of subtelocentric chromosome pair 24 and were congruent with heterochromatic blocks (see Figures 1b and 2c).

## Discussion

The observed 2n = 50 metacentric, submetacentric, and subtelocentric chromosomes of *H. wheatlandii* are characteristic of other Bryconinae [29]–[33], [13]. The absence of telocentric chromosomes may represent an apomorphy within the taxon, despite some variation in karyotypic formulae. This karyotypical structure is shared with species of the genus *Salminus* [34], suggesting a close phylogenetic relationship with Bryconinae, a pattern supported by molecular data [35]–[37]. In this context, karyological evidence also adds support for the existence of the family Bryconidae, as suggested by Oliveira and colleagues [37]. However, from a morphological standpoint, *Salminus* species are still considered to be *incertae sedis* within Characidae [9]. On the other hand, the presence of a metacentric chromosome pair as the largest of the complement in *H. wheatlandii* and other Bryconinae [33] is characteristic of the family Characidae [38], [39].

The presence of pericentromeric and telomeric heterochromatic blocks on opposite arms of the first pair of metacentric chromosomes of *H. wheatlandii* differs from the equilocal pattern observed in other Bryconinae, and thus has great relevance for understanding the karyotypical evolution of this species. Equilocal heterochromatic markings are common in Bryconinae species and considered to be plesiomorphic within this taxon [30]. This pattern has been reported in *Brycon orthotaenia*, *Brycon hilarii*, *Salminus hilarii* [30], and *Brycon amazonicus* [13]. The different pattern observed in *H. wheatlandii* suggests the occurrence of paracentromeric inversions in this species. To the best of our knowledge, this is the first record of paracentromeric inversions in Characidae. Chromosomal inversions are crucial for the reproductive isolation of populations [40] and contribute to the speciation process [41]–[44]. High levels of variability in heterochromatin patterns of *Brycon* were considered to be relevant factors in chromosome evolution and differentiation of the Bryconinae [29], [30], and the presence of large heterochromatic blocks in the first pair of submetacentric chromosomes [13], [29], [30], probably represents a plesiomorphic character state for the subfamily Bryconinae. Based on overall patterns of heterochromatin distribution, Margarido and Galetti Jr. [29] proposed that *Brycon* species might be separated into two groups: the first characterised by predominantly pericentromeric heterochromatin in submetacentric chromosomes; and the second diagnosed by telomeric markings in metacentric chromosomes. *Henochilus wheatlandii* exhibits a third pattern, with markers prevalent in the pericentromeric region of subtelocentric chromosomes. This characteristic seems to be an autapomorphy for this taxon.

The physical congruence of 18S probe sites, CMA_3_ fluorescence, and Ag-positive NOR locations in *H. wheatlandii* was similar to patterns observed for *Brycon falcatus*, *Brycon cephalus*, *B. hilarii*, *Brycon orbignyanus*, *B. orthotaenia*, *Brycon insignis*, and *B. amazonicus* [31], [13], indicating the presence of a single 18S rDNA locus in Bryconinae. However, the 18S rDNA site occurred on the subtelocentric chromosome pair in *H. wheatlandii*, instead of the first pair of submetacentric chromosomes as in all *Brycon* species. Also, the 5S rDNA site in *H. wheatlandii* was found in a pair of subtelocentric chromosomes, whereas in *Brycon* species the 5S rDNA occurs in submetacentric chromosomes [32], [13]. These differences indicate a long independent karyotypic evolutionary history of *H. wheatlandii*. *Henochilus wheatlandii* also showed congruence of the 5S rDNA sites with heterochromatic blocks, which is a common characteristic of *Brycon* [29], [32] suggesting that this is plesiomorphic trait for Bryconinae.

**Figure 3:**
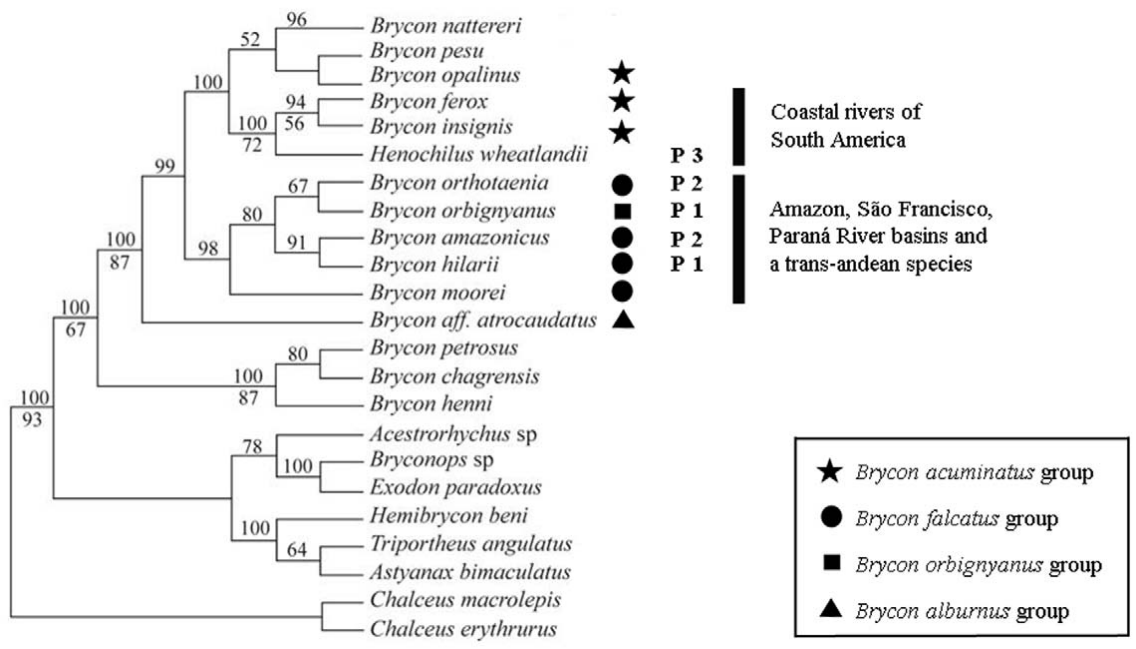
Molecular data-based phylogenetic hypothesis for some Bryconinae. Numbers above branches are Bayesian posterior probabilities expressed in percentage, numbers below branches indicate maximum likelihood analyses bootstrap values (modified from Hilsdorf and colleagues [10]). Morphological groups proposed by Howes [45]: stars for *Brycon acuminatus*, circles for *Brycon falcatus*, squares for *Brycon orbignyanus*, and a triangle for *Brycon alburnus*. P1 and P2, heterochromatic patterns proposed by Margarido and Galetti [29]. P3 is a new pattern recorded in *Henochilus wheatlandii*. Bars indicate geographical distribution.

The occurrence of three heterochromatic karyotype patterns in Bryconinae is partially congruent with some phylogeographic clades based on data from the mitochondrial 16S rDNA gene [10] and is also consistent with the morphological groups proposed by Howes in 1982 [45] (see Figure 3). Based on molecular data, Hilsdorf and colleagues [10] obtained some well-supported molecular clades. Within the eastern coastal clade, *H. wheatlandii* is the sister species of *Brycon insignis* and *Brycon ferox*. The C-banding patterns of *B. insignis* were interpreted by Margarido and Galetti [29] as an instance of the second heterochromatic pattern [29]. However, this species, like *H. Wheatlandii*, has a high number of subtelocentrics with heterochromatic blocks and the absence of equilocal heterochromatic blocks in the first chromosome pair, which might be the result of a paracentromeric inversion in the ancestor of both species. Differences in the marking locations of 5S rDNA sites sets *H. wheatlandii* apart from all other Bryconinae. Whereas in this species 5S rDNA markings were located in a small subtelocentric chromosome, the probe only occurs in submetacentric chromosomes in species endemic to other basins: *Brycon orthotaenia* (São Francisco River), *B. hilarii* (Paraguay River), *B. orbignyanus* (La Plata River), *B. cephalus* (Amazon drainage), and *Brycon* sp. (Araguaia River, a specimen collected by Wasko [32]). 5S rDNA markings on submetacentrics also characterise *Brycon amazonicus* (from the Amazon and Orinoco basins) and *B. falcatus* (from the Amazon, Orinoco, and Guyana/Suriname basins) [32], [46]. The presence of 5S rDNA in two pairs of submetacentric chromosomes in *B. insignis* and a small subtelocentric chromosome in *H. wheatlandii* suggests a high level of variation of this ribosomal gene in species that occur in eastern Brazil. This may be the result of a relatively longer geological isolation of the eastern basins compared with the other drainage areas of the Neotropical region, which have a complex history of fragmentation and connections [47].

Castro and colleagues [1] reviewed the historical changes of the systematic position of *H. wheatlandii*. The species was initially considered by Garman [3] as closely related to *Tetragonopterus* and *Scissor*. Later, Eigenmann and Myers [2] viewed *H. wheatlandii* as “an aberrant member of the Tetragonopterinae” and Géry [7] added the species to the Cheirodontinae. Malabarba [8] redefined the Cheirodontinae and placed it in *incertae sedis* condition within Characidae. Currently, due to the presence of premaxillary large teeth and symphysial dentary teeth, the species is considered morphologically within Bryconinae [9]. Molecular-based studies show that *H. wheatlandii* is closely related to *Brycon insignis* [10], [37] and *Brycon ferox* [10], suggesting the need to include *Henochilus* within *Brycon*. Aside from this caveat, the inclusion of *H. wheatlandii* within *Brycon* will keep this species as a clear example of adaptive radiation [48].

The unique cytogenetic features of *H. wheatlandii* compared to other species of Bryconinae from continental biogeographical units suggest this species (possibly together with other species from the eastern drainage areas) is a separate phylogenetic unit that faces high extinction risks.

## Supporting Information

Figure S1 **Chromosome spread from karyotypes presented in this work.** Conventional staining (Giemsa) (a); C-banding protocols (b); Ag-NOR banding protocols (c); chromomycin A3 (d), and fluorescent in situ hybridisation using 18S (pink) and 5S (green) probes (e).
(TIF)
