# Supplementary figures and images for: The Unique Karyotype of Henochilus wheatlandii, a Critically Endangered Fish Living in a Fast-Developing Region in Minas Gerais State, Brazil
Source: PLoS One. 2012 Jul 27;7(7):e42278. doi: 10.1371/journal.pone.0042278 (PMC3407097; doi:10.1371/journal.pone.0042278)

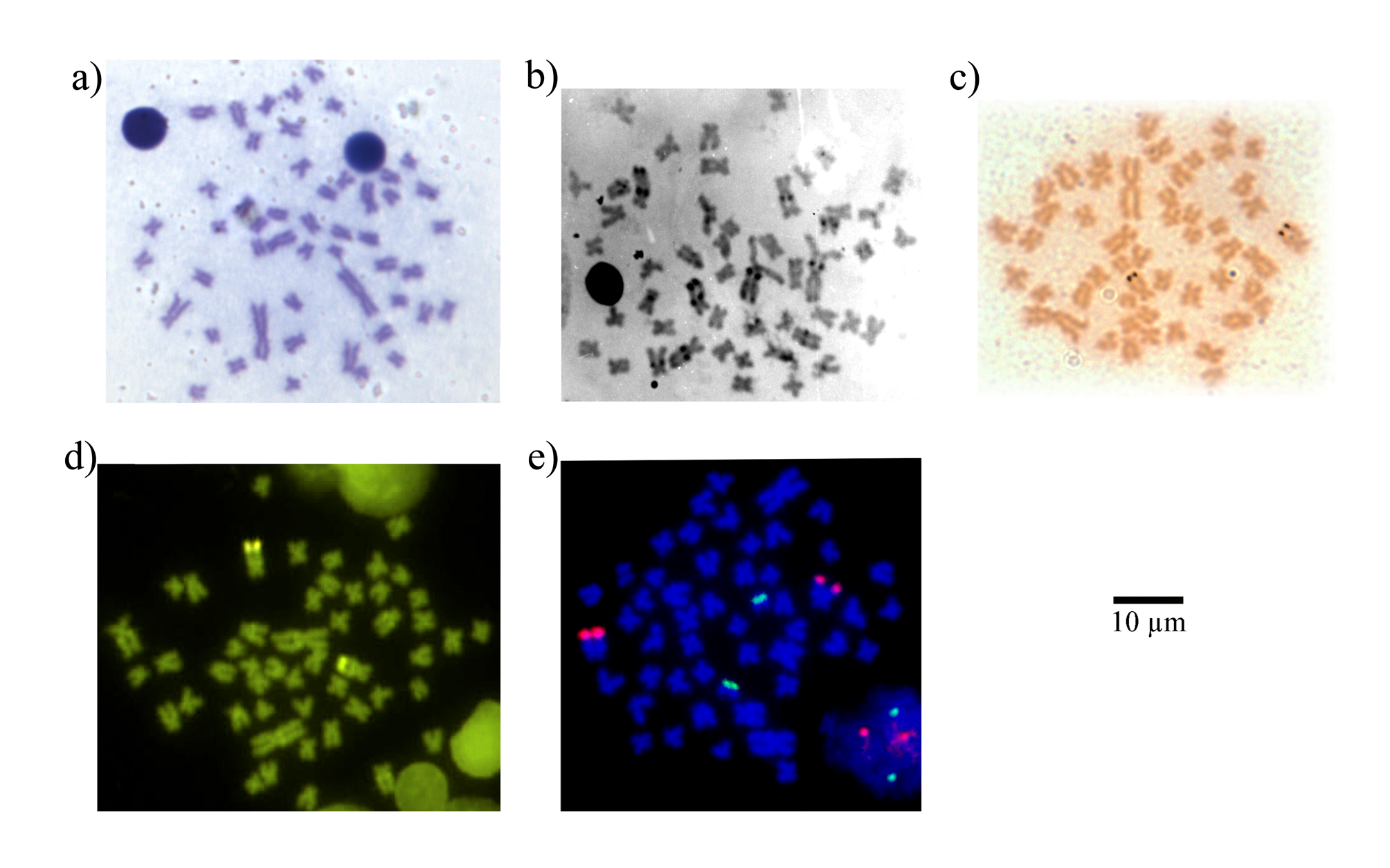

Supplement: Figure S1 — Chromosome spread from karyotypes presented in this work. Conventional staining (Giemsa) (a); C-banding protocols (b); Ag-NOR banding protocols (c); chromomycin A3 (d), and fluorescent in situ hybridisation using 18S (pink) and 5S (green) probes (e). (TIF) [file pone.0042278.s001.tif]
